# Persistent Left Superior Vena Cava: Clinical and Procedural Challenges

**DOI:** 10.7759/cureus.81931

**Published:** 2025-04-08

**Authors:** Rishabh Kasarla, Jordan Ditchek, Mark Elias, Pranav S Tadepalli, Mohammadali M Shoja

**Affiliations:** 1 Medical Education, Nova Southeastern University Dr. Kiran C. Patel College of Allopathic Medicine (NSUMD), Fort Lauderdale, USA; 2 Medical Education, Nova Southeastern University Dr. Kiran C. Patel College of Osteopathic Medicine (KPCOM), Fort Lauderdale, USA

**Keywords:** bilateral superior vena cava, left sided superior vena cava, single versus two veins, superior vena caval duplication, vascular malformations

## Abstract

Persistent left superior vena cava (PLSVC) is an under-recognized thoracic venous anomaly. It typically drains venous blood from the left arm and neck, and often terminates in the coronary sinus on the right side of the heart. Although often asymptomatic, PLSVC is frequently discovered incidentally during imaging or interventional procedures. This anatomical variation can pose challenges during procedures such as venous catheterization and pacemaker placement, due to its atypical drainage route. This report presents a case of PLSVC in a patient who underwent placement of a peripherally inserted central venous catheter through the left arm, aiming to raise awareness of this anatomical variation among healthcare professionals. It includes a brief review of the embryology and anatomy of PLSVC, emphasizing that improved recognition and understanding of this venous anomaly can enhance diagnostic accuracy and procedural safety.

## Introduction

A persistent left superior vena cava (PLSVC) is an additional left-sided superior vena cava (SVC) that originates at the junction of the left subclavian and internal jugular veins and typically drains into the right atrium via the coronary sinus. This thoracic venous anomaly has a reported incidence of 0.5%-2% in the general population and up to 10% in patients with congenital heart disease [1]. The prevalence of PLSVC is significantly higher in congenital conditions such as double outlet right ventricle, tetralogy of Fallot, atrial septal defects, and patent ductus arteriosus [2]. This association arises because both PLSVC and congenital heart defects stem from abnormal embryological development of related vascular structures. PLSVC arises from improper remodeling of the primitive circulatory channels during weeks 3-5 of embryonic development [3]. In early embryogenesis, venous return is facilitated by three paired veins: the vitelline, umbilical, and cardinal veins. The normal formation of a right-sided SVC occurs through the regression of the left common and anterior cardinal veins. However, when these veins fail to regress, a left-sided SVC persists [4].

In the absence of congenital heart disease, PLSVC is often asymptomatic due to the lack of significant right-to-left shunting. It is usually discovered incidentally during imaging studies, cardiothoracic surgery, or autopsy [1]. In approximately 10% of cases, PLSVC drains into the left atrium, either through the left pulmonary veins or an unroofed coronary sinus. Depending on the degree of right-to-left shunting, patients may remain asymptomatic or develop symptoms such as fatigue, syncope, exercise intolerance, or cyanosis [4]. In rare cases, PLSVC can occur in the absence of a right-sided SVC. This condition, known as isolated PLSVC, has an incidence of only 0.07%-0.13% in individuals with congenital heart disease [5].

In this report, we describe a patient with PLSVC who received a venous catheter, with post-procedural imaging revealing an atypical catheter trajectory. This unusual catheter trajectory may be unanticipated by healthcare professionals, potentially causing confusion that could prompt unnecessary diagnostic evaluations or interventions. The aim of this report is to raise awareness of PLSVC, to prevent misinterpretation during radiological assessments, and to facilitate appropriate patient management.

## Case presentation

**Figure 1:**
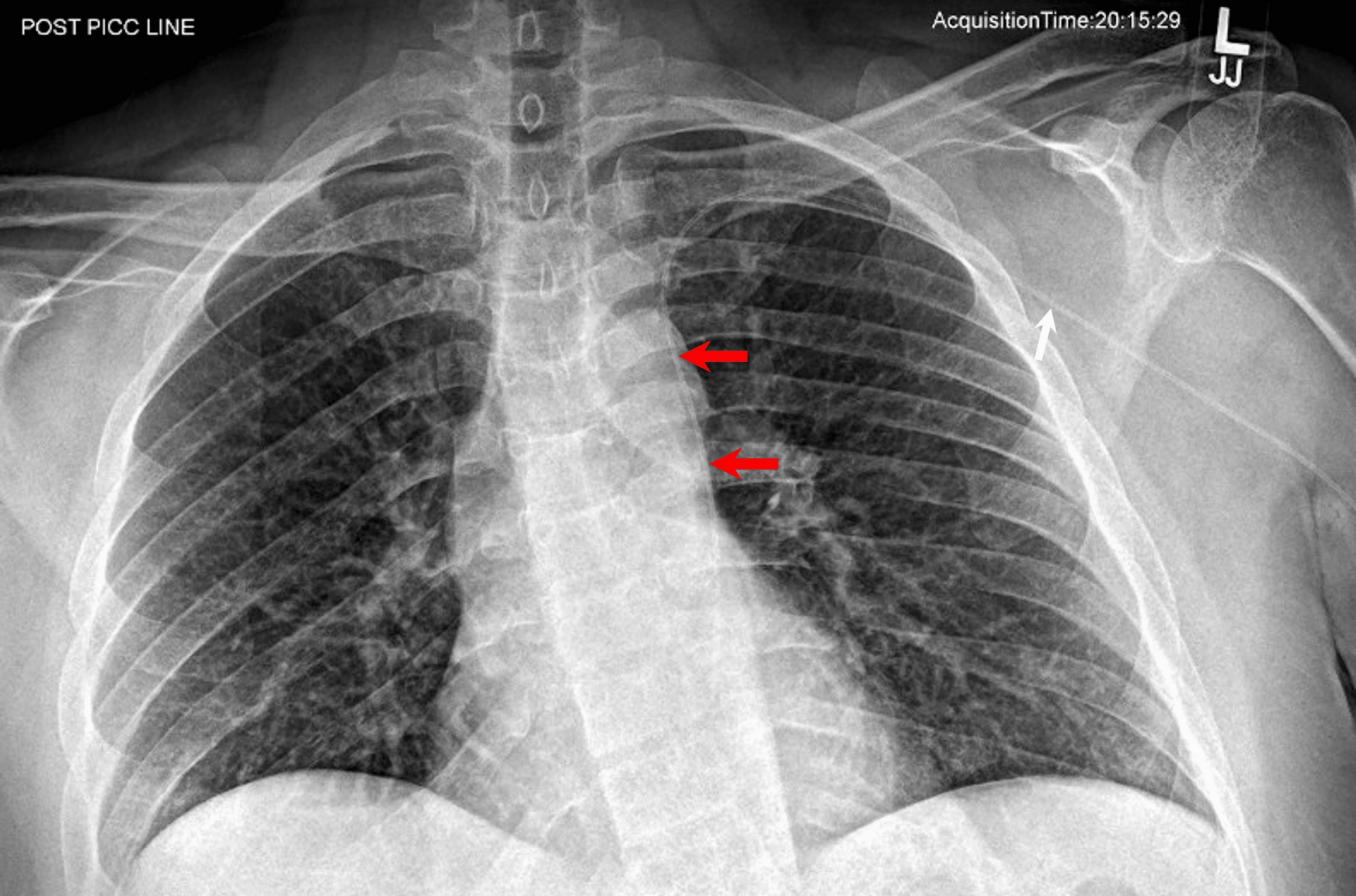
Anteroposterior X-ray of the chest Note that the peripherally inserted central catheter (red arrows) in the left upper extremity does not cross to the right of midline to the expected location of the superior vena cava. It courses centrally to the expected location of the left subclavian vein, but then turns inferiorly into the left side of the mediastinum.

A patient undergoing long-term intravenous treatment received a peripherally inserted central catheter (PICC) through the left upper extremity. A post-procedural X-ray demonstrated the PICC line coursing centrally through the left subclavian vein, then turning inferiorly towards the left side of the mediastinum (Figure 1).

**Figure 2:**
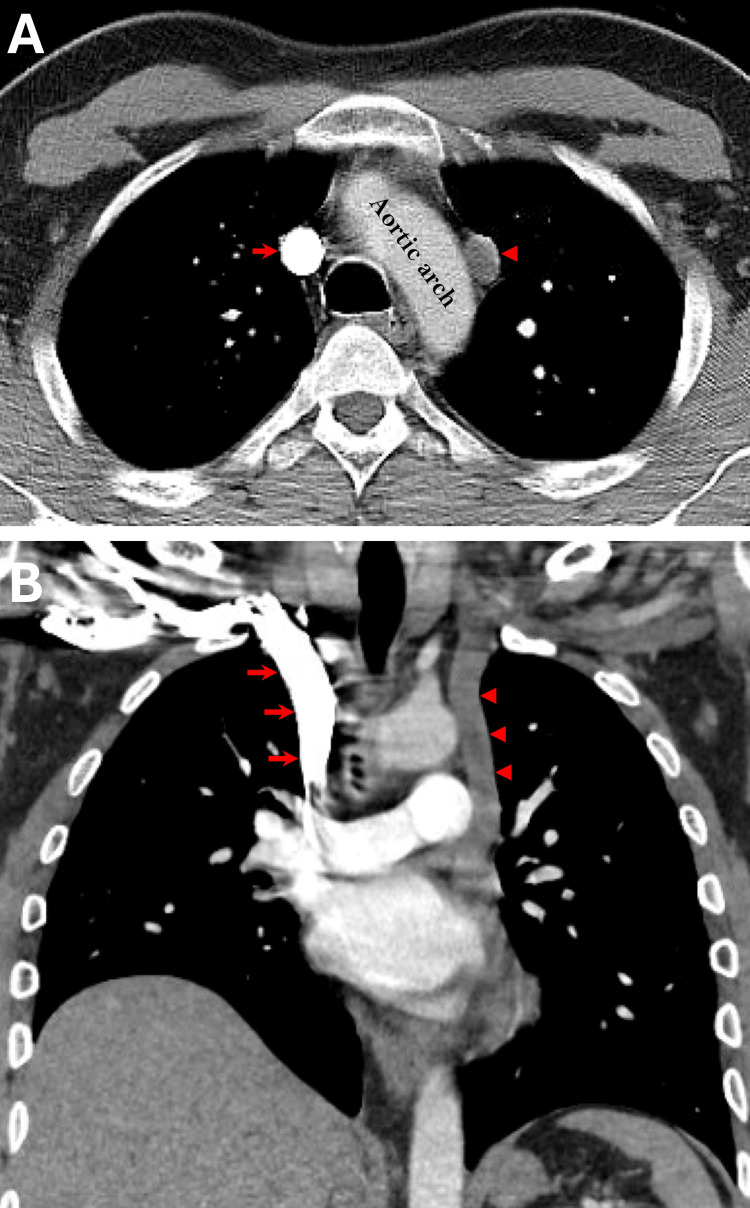
Computed tomography Axial (A) and coronal (B) images from a contrast-enhanced computed tomography scan of the chest demonstrate a tubular structure on the left side of the mediastinum (red arrowheads), with a parallel course to the normal right-sided superior vena cava (red arrows), consistent with the left-sided component of a duplicated superior vena cava. Note the dense opacification of the right-sided SVC due to injection of the intravenous contrast via a right upper extremity vein in this patient. The left-sided SVC contains unopacified venous blood returning from the left upper extremity. The aortic arch is partially opacified on this scan, acquired during an early phase of the contrast administration. SVC, superior vena cava

A computed tomography (CT) scan demonstrated a tubular structure on the left side of the mediastinum, adjacent to the aorta, running parallel to the right-sided SVC, suggestive of a PLSVC. There was no evidence of cardiac chamber enlargement in the imaging. Contrast administration via the patient’s right upper extremity vein opacified the right-sided SVC, whereas the left-sided SVC, draining venous blood from the left upper extremity, remained unopacified (Figure 2).

## Discussion

Misinterpreting aberrant vascular anatomy can result in misplaced lines, inaccurate assessments of catheter placement, and unwarranted line removals or adjustments, all of which can negatively impact patient care. To avoid unnecessary catheter repositioning, healthcare providers should recognize that an abnormal catheter trajectory on X-ray imaging may reflect an anatomical variation rather than a placement error. This has been reported in cases where left-sided central catheter insertions terminate in the coronary sinus or left atrium [6,7]. In such situations, placing the central catheter through the right upper extremity can ensure it follows the typical path through the right-sided vena cava [8]. Additionally, the radiological appearance of a PLSVC may be mistaken for other conditions, such as an enlarged lymph node, an aortic arch aneurysm, a vascular tumor, or thrombosis. Such misinterpretations can lead to unnecessary diagnostic tests, treatments, or procedures.

Zhou et al. contend that PLSVC is an under-recognized entity, owing to its typically asymptomatic nature in cases with right atrial drainage and the routine clinical preference for cannulating the right internal jugular or subclavian vein rather than left-sided veins [9]. Awareness of the possibility of a PLSVC, particularly given its prevalence of up to 10% in patients with congenital heart disease, is crucial to prevent avoidable complications during intravascular procedures. Serious complications, including cardiac arrhythmias, coronary sinus thrombosis, cardiac tamponade, and cardiac arrest, have been reported in association with PLSVC catheterization [9-11]. Nonetheless, Zhou et al. documented three successful PLSVC cannulations for hemodialysis catheter placement, intravenous fluid therapy, and chemotherapy administration without complications [9]. Catheterization of the PLSVC for hemodialysis has been demonstrated to be safe under the following conditions: (1) adequate drainage of the PLSVC into the right atrium, confirmed by echocardiography or angiography; (2) patency of the left innominate vein, verified by CT imaging; (3) electrocardiogram (ECG) monitoring shows no arrhythmias are provoked during the catheterization; and (4) venous blood confirmed through blood gas analysis [12,13].

In up to 20% of individuals with PLSVC, the right SVC is absent [14]. Schummer et al. documented a case of PLSVC with an absent right SVC, where single-chamber pacemaker implantation was attempted via the left subclavian vein [14]. The pacemaker lead was successfully advanced through the PLSVC and coronary sinus into the right atrium and positioned in the right ventricle. Azocar et al. reported an interesting case involving an ICU patient who underwent placement of a central line via the right subclavian vein [6]. Post-procedure chest X-ray revealed the catheter crossing the midline and extending toward the left hilar region, raising initial concerns about procedural complications, such as vein perforation or intra-aortic catheter placement. However, further evaluation with CT confirmed the absence of a right SVC and the presence of a PLSVC draining into the right atrium via an enlarged coronary sinus. PLSVC is rarely associated with an unroofed coronary sinus, a condition characterized by partial or complete absence of the common wall between the coronary sinus and the left atrium [15,16]. While this combination may remain asymptomatic, it can lead to complications such as right ventricular failure, paradoxical embolism, or cyanosis [15].

Brief review of anatomy and embryology

**Figure 3:**
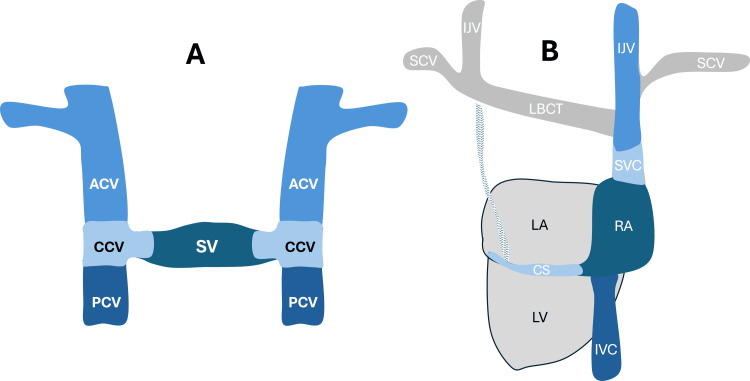
Embryogenesis of thoracic venous system (A) Early embryogenesis of the coronary sinus and cardinal veins; (B) Fully developed adult venous system. Image credit: Mohammadali M. Shoja ACV, anterior cardinal vein; CCV, common cardinal vein; CS, coronary sinus; IJV, internal jugular vein; IVC, inferior vena cava; LA, left atrium; LBCT, left brachiocephalic trunk; LV, left ventricle; PCV, posterior cardinal vein; RA, right atrium; SCV, subclavian vein; SV, sinus venosus; SVC, superior vena cava

The embryogenesis of the thoracic venous system (Figure 3) and PLSVC has been elegantly reviewed by Saremi et al. [17] and Verma et al. [18].

**Figure 4:**
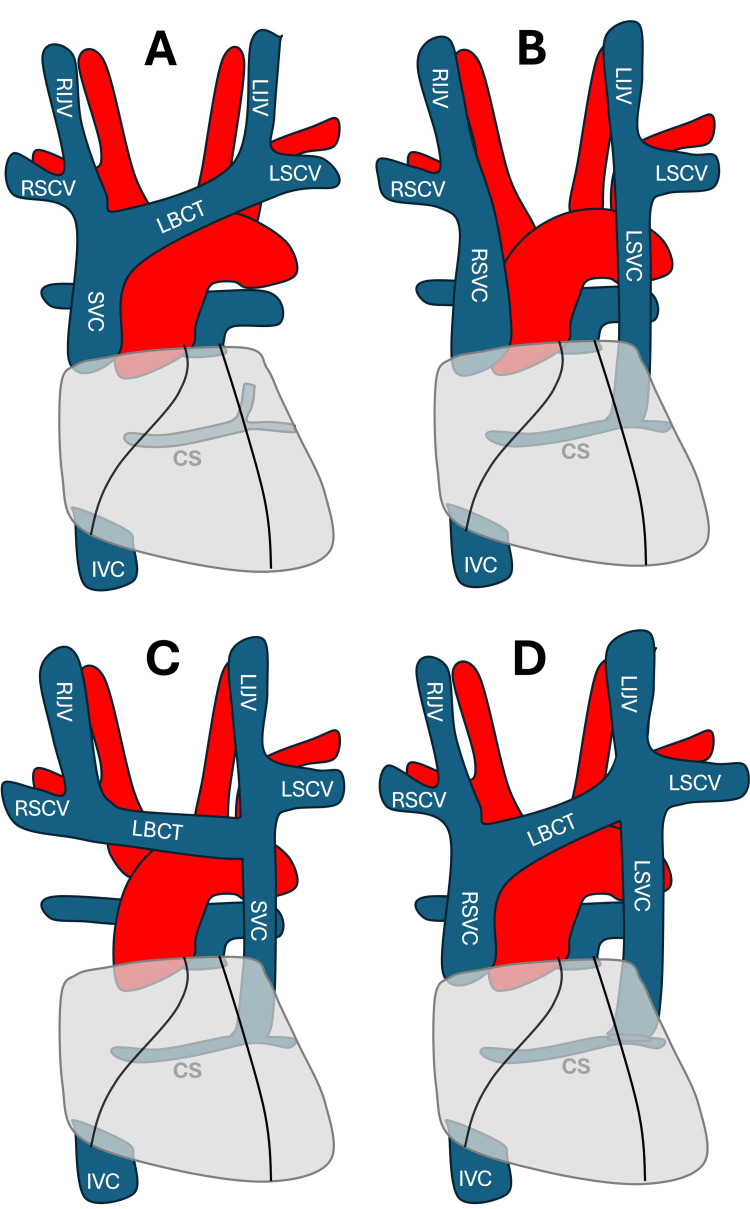
Morphological variants and anomalies of superior vena cava (SVC) (A) Normal right-sided SVC; (B) Persistent left SVC without communication with the right SVC; (C) Isolated left-sided SVC; (D) Persistent left SVC with a venous connection to the right SVC. Image credit: Mohammadali M. Shoja CS, coronary sinus; IVC, inferior vena cava; LBCT, left brachiocephalic trunk; LIJV, left internal jugular vein; LSCV, left subclavian vein; LSVC, left superior vena cava; RIJV, right internal jugular vein; RSCV, right subclavian vein; RSVC, right superior vena cava; SVC, superior vena cava

In a nutshell, the embryonic thoracic venous system begins with two anterior cardinal veins and two posterior cardinal veins that drain blood from the cranial and caudal regions of the developing embryo. These veins converge to form the right and left common cardinal veins, which drain into the ipsilateral horns of the sinus venosus in the developing heart. By the eighth week of gestation, an anastomosis occurs between the right and left anterior cardinal veins, resulting in the formation of the innominate (or brachiocephalic) vein. The cephalic portions of the anterior cardinal veins give rise to the internal jugular veins on each side. The caudal portion of the right anterior cardinal vein and right common cardinal vein develops into the normal right-sided SVC. The right horn of the sinus venosus forms the posterior wall of the right atrium. The left horn of the sinus venosus and the left common cardinal vein form the coronary sinus and the ligament or vein of Marshall (oblique vein of the left atrium). The caudal portion of the left anterior cardinal vein, below the level of anastomosis between the two anterior cardinal veins, typically regresses. However, if this regression does not occur, a PLSVC develops, draining into the coronary sinus in most cases. In such cases, the innominate vein may or may not degenerate. In approximately 60% of individuals with a combined right-sided SVC and PLSVC, the two are connected by the innominate vein [14]. Figure 4 demonstrates various morphological variants and anomalies of the SVC.

The PLSVC descends lateral to the aortic arch, then anterior to the left lung root, before entering the pericardium at the posterior atrioventricular groove, where it drains into the beginning of the coronary sinus [7]. On chest X-rays, it may present as a focal widening of the mediastinum above the left side of the aortic knob [7]. An enlarged coronary sinus is a common hallmark feature of PLSVC and should prompt suspicion of this anomaly when observed in imaging studies [7]. The arrhythmogenic potential of PLSVC is well-documented in the literature [19]. PLSVC may serve as a source of ectopic beats, triggering atrial fibrillation through its anatomical connections to the left atrium near the left pulmonary vein, or via the coronary sinus [20]. In a study of 300 patients with cardiac rhythm disorders who underwent electrophysiological studies or permanent pacemaker implantation, Morgan et al. reported a 4% prevalence of PLSVC, significantly higher than that observed in the general population [19]. The authors hypothesized that embryological anomalies giving rise to PLSVC may also result in abnormalities of the early conduction tissue, which develops in close proximity to the cardinal venous structures.

Procedural safety

Recognizing anatomical variations, such as PLSVC, is essential for ensuring optimal patient management, particularly in terms of surgical outcomes and procedural safety. From a procedural standpoint, awareness of PLSVC is particularly important for cardiologists during pacemaker or defibrillator placement, as the altered anatomy of the vessels and heart increases the risk of lead dislodgement or perforation [21]. In patients with suspected or confirmed PLSVC, accessing the right subclavian vein or internal jugular vein for device placement may help minimize complications. Awareness of PLSVC can help prevent complications during surgeries involving the thoracic cavity and mediastinum. For instance, during retrograde cardioplegia in open-heart surgery, the cardioprotective electrolyte solution may drain into the PLSVC instead of remaining within the coronary sinus and heart tissue, rendering the procedure ineffective for stabilizing the heart [22]. In such cases, clamping the PLSVC has been shown to restore the effectiveness of retrograde cardioplegia [23].

## Conclusions

This case report illustrates an example of a significant, albeit benign, anatomical variation that can lead to misinterpretation of imaging and potential complications during central venous catheterization. A PLSVC is a vascular malformation with a spectrum of presentations, depending on the underlying anatomy. These range from asymptomatic cases, where it presents merely as an anatomical variation, to cases involving right-to-left shunting when the PLSVC drains into the left atrium, cardiac function compromise, or associations with congenital heart defects.
